# Socio-Economic Status and Psychological Well-Being in a Sample of Turkish Immigrant Mothers in Germany

**DOI:** 10.3389/fpsyg.2018.01586

**Published:** 2018-10-04

**Authors:** Ina Fassbender, Birgit Leyendecker

**Affiliations:** Psychology, Ruhr-Universität Bochum, Bochum, Germany

**Keywords:** immigrant mothers, life satisfaction, depression, socio-economic status, Turkish immigrants, maternal education

## Abstract

This study analyzes the relation of socio-economic status and psychological well-being in a sample of 327 Turkish immigrant mothers in Germany. We assessed maternal psychological well-being with the CES-D-10, the Satisfaction with Life Scale, and selected items of the Hassles Scale referring to daily hassles. Mothers' SES was assessed by means of household income and maternal education. The sample has a predominantly low to very low household income. A cluster analysis on maternal education and household income identified three SES-groups: A low-income cluster, a low-education cluster, and a third cluster of mothers who were slightly more advantaged in terms of household income and education. When applying the 10-point criterion of the CES-D-10, the three clusters differed regarding depression. About 40% of the mothers with lowest income and lowest education were depressed, compared to 28% of the more-advantaged cluster. The clusters further differed with respect to daily hassles and life-satisfaction. A higher SES was associated with less daily hassles, a higher life satisfaction, and less depression. This replicates findings of other studies regarding the relation of SES and psychological well-being. A follow-up assessment for about 60% of the mothers after 1 year revealed no changes in the well-being scales for each SES cluster, and a significant multivariate effect of the SES clusters. This suggests that SES is a long-term influential factor on psychological well-being. We discuss our findings in terms of the importance to integrate Turkish immigrant mother into the Germany society and in terms of the importance of maternal psychological well-being for children's positive development.

## Introduction

With close to 3 million people (Bundesamt für Migration und Flüchtlinge, 2016), Turkish immigrants are the largest minority population in Germany. They are from all social strata, but on average, they are the immigrant group with the lowest income, and a poverty risk rate of 36% (Statistisches Bundesamt, 2016). At the same time, education is often quite low: 45% of the Turkish immigrant children grow up with parents who have neither a professional qualification nor a university entrance qualification. This is the lowest quota among immigrants in Germany (Bundesministerium für Bildung und Forschung, 2016). Turkish immigrants can thus be considered an educationally and financially disadvantaged immigrant group in Germany. In this study, we address the question whether these disadvantages have an impact on psychological well-being of Turkish immigrant families. We focus on mothers who have at least one child attending school or kindergarten in Germany. We assume that this phase can be particularly challenging because mothers may deal with potential conflicts between home culture and host culture, especially when they are first generation immigrants and thus have not had access to the German education system themselves. Even though Turkish immigrant families comprise the largest minority group in Germany and their generally low SES is well known, research on the relationship between SES indicators such as income and education as well as other relevant socio-demographic indicators and mothers' psychological well-being is still scarce.

What is the best way to measure advantages or disadvantages in an immigrant sample? In the migration literature, SES is typically assessed by means of (various measures of) income or the economic situation and education, and in some cases by one of those aspects alone. Barger et al. (2009), for example, assess SES based on education, income, employment status and wealth. Wang et al. (2010) use “both objective (education, household income, and personal annual income) and subjective (financial strain) measures for SES” (Wang et al., 2010, p. 448), but no measure of education, while Negy and Woods (1992) asked Mexican-American university students to report only on their parents' education to measure SES. For a sample of Turkish immigrant mother-child dyads in the Netherlands, Emmen et al. (2013) used the two measures family income and parental education to assess SES. Obviously, there are cumulative advantages, which benefit higher educated individuals and individuals with more privileged positions in society (Miech and Shanahan, 2000). This reflects the social impact of an individual's financial and educational resources. It thus seems important to assess SES by both income and education measures and not by one of those measures (e.g., income) alone.

Both income and education are likely to impact well-being (Barger et al., 2009). A higher education, higher financial resources, and thus, a higher SES increase psychological well-being in various respects. In contrast, a low SES is likely to have a negative impact on well-being. For a representative Canadian sample of immigrants and non-immigrants, Wang et al. (2010) identified a low level of education and financial strain as risk factors for depression in working individuals. Zimmerman and Katon (2005) also outline a robust association of financial strain (as assessed with a debts-to-assets ratio) with depressive symptoms. The authors used a US National Survey sample in which the CES-D (Eaton et al., 2004) was applied to assess depression. They also found unemployment to be a risk factor for depression. A study comparing Moroccan and Turkish immigrants in the Netherlands (Gokdemir and Dumludag, 2012), found different associations between income and life satisfaction in the two immigrant groups. In the Moroccan sample, life satisfaction was dependent on absolute income. In the Turkish sample however, the relative income, i.e. their position in the Dutch society, was the key their life satisfaction. This is explained by the better socioeconomic position of Turkish immigrants when compared to Moroccan immigrants in the Netherlands, and suggests that the fulfillment of aspirations (here to receive enough money to live vs. to gain a good position in society which reflects the social aspect of SES) is the key to life satisfaction. In another sample of Turkish immigrants in the Netherlands, Emmen et al. (2013) found higher SES to be correlated with less acculturation stress and less psychological distress. We can thus summarize that a higher SES assessed by means of income and education is likely to have a positive influence on life-satisfaction and psychological well-being, whereas a lower SES can be a risk factor and increases the risk for depression. This relation seems to be independent of the immigrant population and the host country.

The aim of this study is to understand the link between SES (as defined by income and education) and psychological well-being in a sample of Turkish immigrant mothers in Germany. We apply a three-step approach. First, we analyze the study participants' SES by means of income and education in order to uncover variance in SES and to identify SES-subgroups for further analyses within this low-SES sample. In a second step, we analyze whether the mothers in different SES groups differ regarding psychological well-being. In a third step, we examine possible long-term effects of SES on psychological well-being for a subsample reassessed after 1 year. Overall, we expect to replicate the associations between lower SES and lower psychological well-being found in studies in other countries and with different groups of immigrants. However, we also hope to find variance within this generally low SES sample in order to identify potential risk and potential protective factors.

## Methods

### Participants

The study participants were the mothers of Turkish origin children recruited in the industrialized German Ruhr-area for the project “Social Integration of Migrant Children - Uncovering Family and School Factors Promoting Resilience.” The inclusion criterion for those children was that either one of their parents or their grandparents had been born in Turkey. As consequently, the mothers could be first or second generation immigrants, the questionnaires they filled out for the present study were available in both German and Turkish. For those who had difficulties with reading, questionnaires were read (in Turkish by bilingual research assistants) and answered together with the subject in a setting as quiet as possible. The initial sample for this study consisted of *n* = 327 mothers who provided information on their own education and on family income. In the different steps on the analyses, the sample size is reduced because not all mothers answered all question(naire)s. Sample sizes are provided for each step of the analyses.

The families were recruited for longitudinal assessments according to their children's (pre)school grades. They gave written consent for the participation, and received 25€ for each assessment, during which the families were visited at home by trained bilingual research assistants. The data collections started when the children were (a) in the last year of kindergarten, with two reassessments at grades 1 and 2, or (b) in 4th grade, with two reassessments at grades 5, and 6, or (c) in 7th grade, with one reassessment at grade 8. As a consequence, the age-range of the mothers, measured at the first assessment, is relatively wide with 24–59 years (mean age 35.87, SD 5.58, *n* = 323; 11 mothers did not report on their age). Maternal age will thus be controlled for in the analyses.

For the assessment of the *socio-economic status*, mothers were asked to report on their highest education level and on their monthly household income after taxes. The mean monthly net household income was €1,709.50. Only an 8% of the families had a net household income of €3,000 or more. In comparison, the mean net monthly household income in Germany was €2,922, which indicates that the Turkish immigrant families assessed represent a low-income group, especially when considering that their net household income is a net family income. This is further supported by the fact that 35.8% of the mothers (*n* = 117) reported receiving social welfare benefits themselves, and that 18.3% (*n* = 60) reported their partner (i.e., the family's father) received social welfare benefits. Mothers could have received their education either in Turkey or in Germany. Therefore, education levels were transformed according to ISCED-97 education level classifications (UNESCO, 2006). Tables 1 and 2 show the distribution of both net household income and maternal education.

**Table 1:** Household income (net) per month.

**Net household income per month in €**	***N***	**Percentage of total sample**
Up to 1,000	18	5.5
1,000–1,499	70	21.4
1,500–1,999	87	26.6
2,000–2,499	87	26.6
2,500–2,999	39	11.9
3,000 or higher	26	8.0
Total	327	100.0

**Table 2:** Maternal education.

**Highest degree**	***N***	**Percentage of total sample**
No degree	24	7.3
Primary education	78	26.6
Lower secondary education	138	42.2
Upper secondary education	65	19.9
University degree	13	4.0
Total	327	100.0

### Instruments

To assess the *mother's psychological well-being*, we used three instruments, all of which other authors have used for Turkish immigrants in Germany, or Turkish-Germans, before (Ponizovsky et al., 2013; Jaekel et al., 2015; Kohl et al., 2015). The Turkish immigrant mothers were asked to answer 5 items of the Satisfaction With Life Scale (SWLS) (Diener et al., 1985) (α = 0.87 in our sample), 13 items of the Hassles Scale (Kanner et al., 1981) assessing Daily Hassles (DH) (α = 0.89 in our sample), and the CES-D-10 Depression Scale (Andresen et al., 1994; Tatar and Saltukoglu, 2010 for the Turkish version) (α = 0.83 in our sample). The CES-D-10 has a 4-point scale ranging from 0 (less than once a week) to 3 (5–7 times a week). It allows for two measures, given that at least 8 of the 10 items are answered, and that a sum score is calculated. First, an individual is diagnosed as depressed with a sum score of 10 or higher, which creates a categorical variable; second, the sum score can be treated as a continuous variable, with higher scores representing a higher degree of depression. The SWLS has a 7-point scale ranging from 1 (extremely untrue) to 7 (extremely true). Higher scale scores reflect higher life satisfaction. DH are assessed with a 5-point scale ranging from 1 (no hassle) to 7 (big hassle). Thus, smaller scale scores reflect fewer daily hassles.

We run a principal axis factor analysis on all items of the three scales. To maintain the sample sizes as big as possible, pairwise exclusion was used in case of missing responses. Thus, for the single items, the sample sizes vary between 308 and 319 mothers. The Kaiser-Meyer-Olkin measure of sampling adequacy was high (KMO = 0.886). The three scales SWLS, DH and CES-D-10 reappeared in the three factors found. As the mean variance extracted from the three scales (0.414) was bigger than the mean correlation square of the three scales (0.179), discriminant validity can be assumed. To further control for common method bias, we extracted one single factor in a principal axis factor analysis (pairwise exclusion) on all items of the three scales. The factor found explains 25.28% of the variance and the Kaiser-Meyer-Olkin measure of sampling adequacy remained at KMO = 0.886. We may thus assume that our data are not affected by common methods bias.

For hypothesis testing, scale scores (sum scores for SWLS and CES-D-10, means for DH) were only calculated if at least 80% of the items (4 items of the SWLS, 11 items of the DH, 8 items of the CES-D-10) were answered.

## Results

### SES subgroups

In order to identify possible SES-subgroups or SES-variance within our predominantly low-income sample, we conducted a two-step cluster analysis on the families' net household income and the mothers' highest educational degrees. The cluster solution with the lowest BIC (164.5) revealed three almost equally distributed clusters. 109 mothers (33.3% of the sample) were attributed to cluster 1, cluster 2 consists of 97 mothers (29.7%), and cluster 3 consists of 121 mothers (37.0%). The largest cluster's size is 1.25 times the smallest cluster's size. The cluster quality is good with a silhouette of 0.5. The two-cluster and four-cluster solutions yielded BIC = 199.4 and BIC = 164.6, respectively. Despite its similar fit to the three-cluster solution, the four cluster model does not provide equally distributes clusters. Cluster 1 and cluster 3 are equally big than in the three-cluster model, while the 97 mothers of the three-cluster solution cluster 2 are split up into two clusters. Thus, in the four-cluster solution, the biggest cluster size is almost three times the smallest cluster size. Consequently, we rejected the four-cluster solution. In the three-cluster solution, both predictors have important influence on the cluster formation (1.0 for the net household income and 0.75 for the mother's highest educational degree). For the three clusters, Table 3 displays the minimum, the maximum, and the mean of the two cluster predictors, together with the partner's highest level of education, the number of persons living in the household, maternal age, and age of the child involved in the main study. In all three clusters, the mean household sizes, maternal age, and the child's age are comparable. A minimum of two persons in the household reflects there are two single-parent families in clusters 1 and one single-parent family in cluster 3.

**Table 3:** Descriptive Information clusters 1–3.

	**Cluster 1**				**Cluster 2**				**Cluster 3**			
	**Mean**	**Min**.	**Max**.	***N***	**Mean**	**Min**.	**Max**.	***N***	**Mean**	**Min**.	**Max**.	***N***
Net household income per month (€)	1220.20	0.00	2000.00	109	1438.15	0.00	Over 3000.00	97	2347.75	1000.00	Over 3000.00	121
Mother's highest level of education	2.38	2	5	109	0.75	0	1	97	2.40	0	5	121
Partner's highest level of education	2.35	0	5	103	1.85	0	5	92	2.68	0	5	120
Number of persons in household	4.30	2	7	105	4.76	3	7	92	4.66	2	9	121
Age of mother	35.14	24	55	107	36.14	26	56	96	36.31	24	59	120
Age of child involved in main study	8.57	5	15	109	9.25	5	14	97	9.01	5	14	121

*Education levels: 0, no degree; 1, primary education; 2, lower secondary education; 3, upper secondary education; 4, post-secondary education; 5, tertiary education; 6, doctorate*.

Cluster 1 can be described as the *lowest income* cluster (€283.77/month per capita), with low to medium levels of education in both parents. All mothers had at least a lower secondary education. Note that when raised in Germany, it is almost impossible to have no school education, and school education is mandatory until 18 years of age. Thus, very low education levels indicate that the respective individuals are first-generation immigrants from Turkey. This is especially apparent in cluster 2, consisting of mothers who had either no education at all or primary education only. Their partner's education is also lower than in cluster 1, although the discrepancy between paternal and maternal education is higher in cluster 2 than in the other clusters. The mean net household income is slightly higher than in cluster 1 (€302.13/month per capita), but is still very low. Thus, cluster 2 can be described as the *low education* cluster in this sample. In comparison to clusters 1 and 2, cluster *3* is a *more advantaged* cluster (€508.10/month per capita). As in cluster 1, all mothers had at least lower secondary education, but fathers' education was a bit higher than maternal education and almost all fathers in cluster 3 were gainfully employed.

Table 4 provides further sociodemographic information on the families. Although the predictors were maternal education and net household income, the three clusters differ in more relevant aspects. This indicates that income and maternal education are relevant variables to describe SES. While almost all the participants' kindergarten or school-aged children involved in the main study were born in Germany, slightly more than 70% of the mothers in clusters 1 and 3 were born in Turkey. However, about one third of the mothers in both clusters migrated to Germany as a child, 21.1% (cluster 1) and 15.7% (cluster 3) in early childhood. With more than 80% in cluster 1, the mothers' partners were also predominantly born in Turkey. In cluster 2, in line with the low education levels, more than 90% of the mothers are first generation immigrants, and more than 70% migrated to Germany being older than 15 years. Of their partners, almost three quarters were born in Turkey. The small differences found for education levels of the mothers' partners between the *lowest income* cluster 1 and the *more advantaged* cluster 3 are also reflected in the partners' professional qualifications. While in both clusters, about 43% of the mothers have a professional qualification, almost half of the partners in cluster 1 have a professional qualification, but in cluster 3, it is 62.07% of the partners. Education and professional qualification are linked to gainful employment of more than 95% of the partners in cluster 3. Although maternal education hardly differs between clusters 1 and 3, almost half of the mothers in cluster 3 are gainfully employed (which does not mean they earn much, but they work), while in cluster 1, less than 25% of the mothers are gainfully employed. This pattern can be considered an explanation for the fact that in cluster 1, 42.06% of the mothers (and almost 30% of their partners, which is more than the 70% of the parents in cluster 1) receive social welfare benefits. The percentage of recipients of social welfare benefits is very similar in cluster 2. Here, less, but still over a third of the mothers receive social welfare benefits. In cluster 3, social welfare benefit reception is the lowest of the sample, but still amounts to over a third of the parents, which reflects the elevated necessity for social support in this Turkish immigrant family sample. This underlines the sample's overall low SES.

**Table 4:** Further sociodemographic information on the SES-clusters.

	**Cluster 1**		**Cluster 2**		**Cluster 3**	
	**%**	***N***	**%**	***N***	**%**	***N***
Child was born in Germany	99.00	108	95.00	94	100.00	120
Mother was born in Turkey	71.30	108	90.43	94	70.83	120
Partner was born in Turkey	82.24	107	74.19	93	81.67	120
Mother migrated to Germany before age 7	21.10	104	3.10	91	15.70	113
Mother migrated to Germany after age 15	34.90	104	71.10	91	31.40	113
Mother has a professional qualification	42.31	104	12.63	95	43.70	119
Partner has a professional qualification	49.46	93	38.64	91	62.07	116
Mother is gainfully employed	23.58	106	15.79	95	47.50	120
Partner is gainfully employed	61.86	97	64.37	87	95.83	120
Mother receives social welfare benefits	42.06	107	36.84	91	30.83	120
Partner receives social welfare benefits	29.29	99	31.03	87	3.33	120

### Participants' depression

As outlined, a higher socio-economic status is a protective factor for depressive disorders. The sample of Turkish immigrant mothers is predominantly of low-SES, and thus at a higher risk for depressive disorders. We obtained CES-D-10 scale scores for 99 out of the 109 mothers in cluster 1, for 96 out of the 97 mothers in cluster 2, and for 115 out of the 121 mothers in cluster 3. In cluster 1, the *lowest income* cluster, 39.4% of the mothers obtained scale scores of 10 or higher, and can thus be considered as depressed. In cluster 2, the *low education* cluster, 43.3% of the mothers obtained scale scores of 10 or higher, and in cluster 3, the *more advantaged* cluster, 28.1% of the mothers obtained scale scores of 10 or higher. An univariate ANOVA with maternal age as a covariate reveals a significant interaction effect between the CED-D-10 categorical variable and the SES-clusters [*F*_(2, 305)_ = 3.41, *p* = 0.034, η_*p*_2 = 0.022]. A *post-hoc* Tukey test reveals that the depression rate of cluster 3 differs significantly from the depression rate of cluster 1 (*p* = 0.46) while the depression rate of cluster 2, which is in-between the other two, does not differ significantly from the rates of the other two clusters. There was no interaction with maternal age [*F*_(1, 305)_ = 0.17, *ns*.]. These results indicate that many Turkish immigrant mothers suffer depressive symptoms. They also confirm that low SES is linked to depression with lower SES being associated with a higher depression prevalence. Thus, a higher SES is a protective factor here.

### SES and psychological well-being

**Figure 1:**
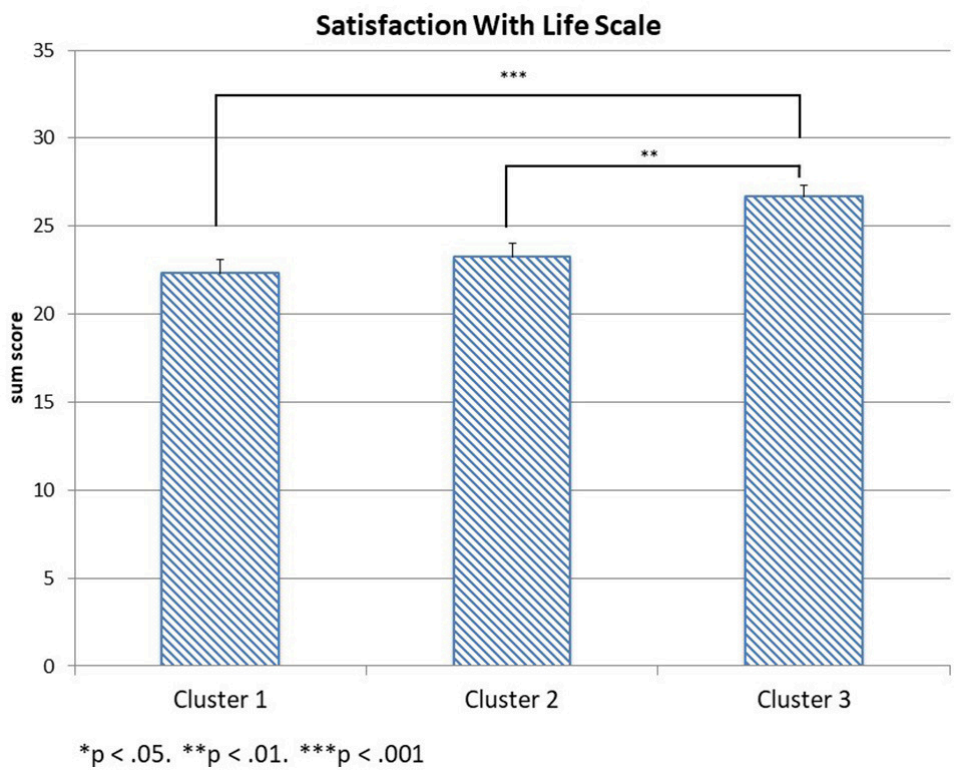
Satisfaction With Life Scale scores in the three clusters.

**Figure 2:**
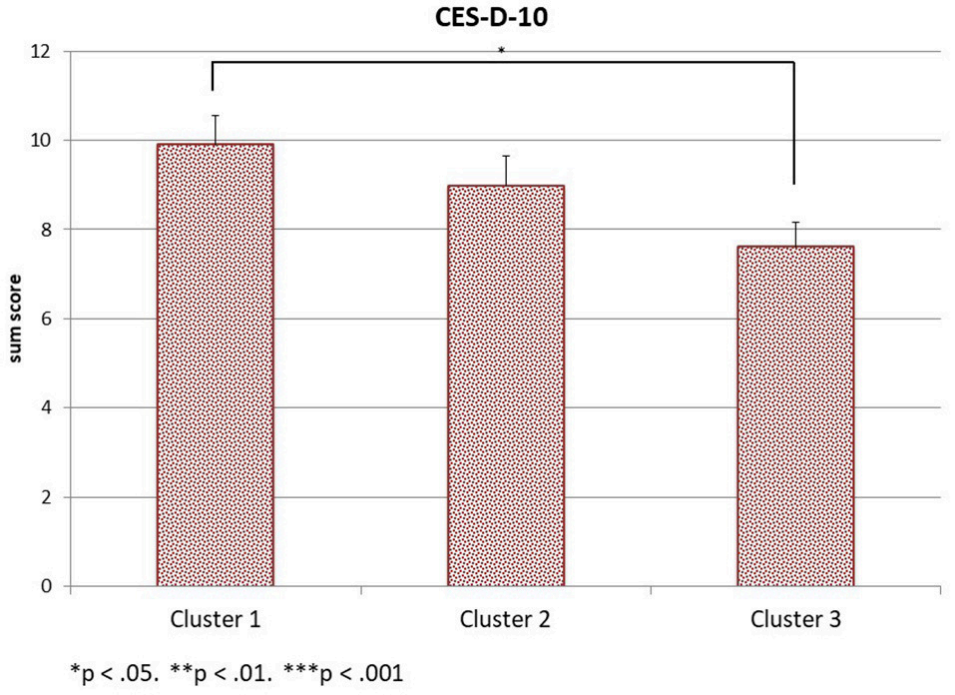
CES-D-10 in the three clusters.

**Figure 3:**
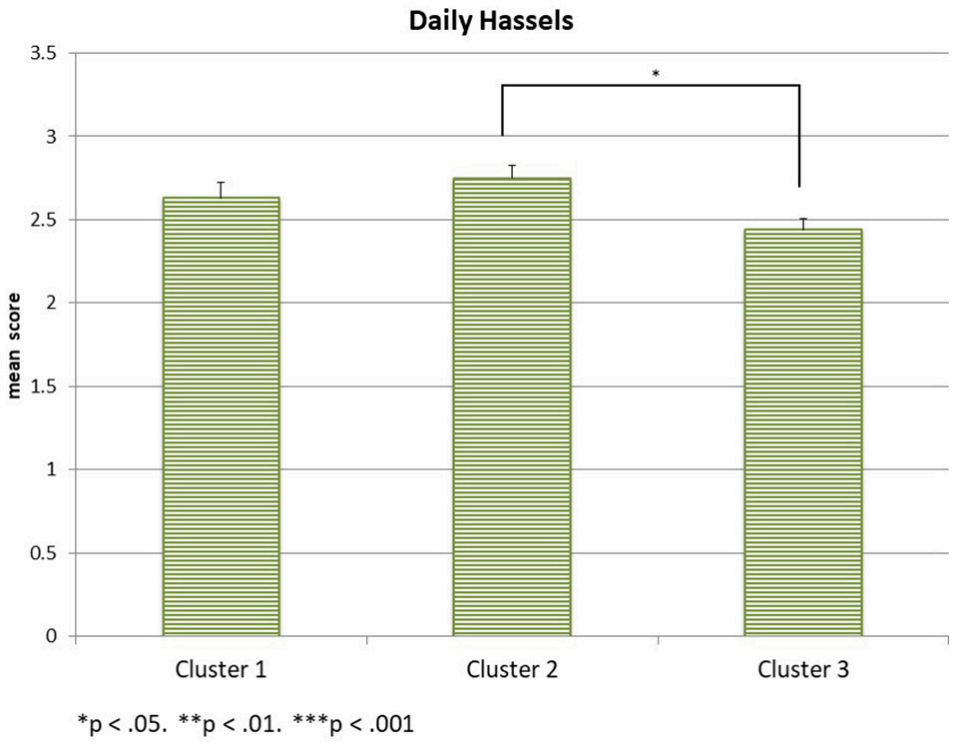
Daily Hassels in the three clusters.

This analysis focuses on whether the three psychological well-being scales (SWLS, CES-D-10 sum score, and DH) differ between the three SES-clusters at the first time of assessment. The three scale scores are the dependent variables in a MANOVA, the SES-cluster is the between-subjects factor, and maternal age is again a covariate. The sample sizes are 95 subjects in cluster 1, 93 subjects in cluster 2, and 112 subjects in cluster 3. The multivariate analysis reveals a significant interaction effect for the SES clusters [*Wilks-*Λ = 0.913, *F*_(6, 588)_ = 4.55, *p* < 0.001, η_*p*_2 = 0.044] with the multivariate outcome, but no interaction effect for maternal age [*Wilks-*Λ = 0.977, *F*_(3, 294)_ = 2.29, *ns*.]. The between-subject effects indicate significant univariate effects of the three clusters on all three psychological well-being scales [*F*_(2, 296)_ = 10.98, *p* < 0.001, η_*p*_2 = 0.069 for SWLS, *F*_(2, 296)_ = 3.87, *p* = 0.022, η_*p*_2 = 0.026 for the CES-D-10 sum score and *F*_(2, 396)_ = 3.74, *p* = 0.025, η_*p*_2 = 0.025 for DH^1^]. *Post-hoc* Tukey tests reveal that the mean SWLS value of cluster 3 (*mean* = 26.67, *SD* = 6.99) differs significantly from the mean SWLS value of cluster 1 (*mean* = 22.32, *SD* = 7.46) at *p* < 0.001 and from the mean SWLS value of cluster 2 (*mean* = 23.24, *SD* = 7.71) at *p* = 0.003 as displayed in Figure 1. Cluster 1 and cluster 2 have comparable life satisfaction values. The CES-D-10 scale scores displayed in Figure 2 differ significantly only between cluster 3 (*mean* = 7.62, *SD* = 5.85) and cluster 1 (*mean* = 9.91, *SD* = 6.32) at *p* = 0.02. The mean CES-D-10 in cluster 2 is 8.99 and does not differ significantly from the means of the other two clusters. Regarding Daily Hassles, displayed in Figure 3, the scale score means of clusters 2 (*mean* = 2.75, *SD* = 0.71) and cluster 3 (*mean* = 2.44, *SD* = 0.73) differ significantly at *p* = 0.031 while the scale score mean of cluster 1 (*mean* = 2.63, *SD* = 0.94) does not differ significantly from the scale score means of the other two clusters. Overall, mothers in the *more advantaged* cluster 3 have a higher life satisfaction, less daily hassles and the lowest depression scores of our sample. Although the *low education* cluster 1 shows the highest depression score of the sample, while the *low income* cluster 2 has the lowest life-satisfaction and the highest daily hassles rate of the sample, the two clusters do not differ significantly on the three scales.

### Influence of SES on psychological well-being after 1 year

Based on 1-year follow-up data, which was available (with the same 80% criteria for all scales) for 63 mothers in cluster 1, for 44 mothers in cluster 2, and for 79 mothers in cluster 3, this analysis controls for changes in the three psychological well-being scales in the different SES clusters. At both measurement points, the three well-being scales are mostly intercorrelated in all clusters, as depicted in Table 5. Higher scale scores stand for higher life satisfaction, higher depression and more hassles, respectively. Thus, correlations between SWLS and the other two scales are negative. Mothers who did not participate in the repeated assessment (with their children) had a lower education [*t*_(1, 301)_ = 3.18, *p* = 0.002^2^, *mean*_(remaining)_ = 2.04, *mean*_(dropout)_ = 1.61], more children [*t*_(1,246)_ = −2.03, *p* = 0.043, *mean*_(remaining)_ = 1.69, *mean*_(dropout)_ = 1.93], and had migrated to Germany at a later age [*t*_(1, 208.49)_ = −2.87, *p* = 0.005 *mean*_(remaining)_ = 14.64 years, *mean*_(dropout)_ = 17.78 years]. Thus, it is not surprising that relatively to the SES-cluster sizes, dropout is highest in the *low education* cluster 2. There were no differences between mothers who participated in the second assessment and who did not with regard to their age, the age of their partners, their partners' education, or in any of the scales used to assess psychological well-being. At the cluster level, in only cluster 1 do differences between mothers who reassessed and not-reassessed remain, namely the effect for migration age [*t*_(1, 66)_ = −2.81, *p* = 0.006, *mean*_(remaining)_ = 10.92 years, *mean*_(dropout)_ = 17.13 years], and the effect of number of children [*t*_(1, 75)_ = −2.28, *p* = 0.026, *mean*_(remaining)_ = 1.38, *mean*_(dropout)_ = 1.74].

**Table 5:** Intercorrelations of the three psychological well-being scales at the first assessment and after 1 year.

	**Cluster 1**				**Cluster 2**				**Cluster 3**			
	**T1**		**T2**		**T1**		**T2**		**T1**		**T2**	
	***r***	***N***	***r***	***N***	***r***	***N***	***r***	***N***	***r***	***N***	***r***	***N***
SWLS and CES-D-10	−0.54^***^	96	−0.48^***^	69	−0.41^***^	94	−0.43^**^	46	−0.30^**^	113	−0.44^***^	83
SWLS and DH	−0.54^***^	96	−0.40^**^	69	−0.10	94	−0.44^**^	46	−0.31^**^	113	−0.42^***^	83
CES-D-10 and DH	0.67^***^	96	0.59^***^	69	0.41^***^	94	0.57^***^	46	0.45^***^	113	0.32^**^	83

** *p < 0.01*,

*** *p < 0.001*.

In a MANOVA with repeated measures, the within-subject factor is time (i.e., the two measurement points), the SES-clusters are between-subject factors, and maternal age at the first assessment is a covariate. The analysis reveals a significant multivariate interaction effect of SES-clusters [*Wilks-*Λ = 0.879, *F*_(6, 360)_ = 3.98, *p* < 0.001, η_*p*_2 = 0.062], and a significant multivariate interaction effect of maternal age [*Wilks-*Λ = 0.933, *F*_(3, 180)_ = 4.31, *p* = 0.006, η_*p*_2 = 0.067]. The regression coefficients only show age differences for DH [ß = −0.03, *t*_(182)_ = −2.80, *p* = 0.006 for time 1 and ß = −0.02, *t*_(182)_ = −2.10, *p* = 0.037 for time 2], indicating that older mothers have less daily hassles than younger mothers at both measurement points. There is no multivariate effect of measurement point [*F*_(1, 182)_ = 0.38, *ns*.], no multivariate interaction with the clusters [*F*_(2, 182)_ = 1.26, *ns*.], and no multivariate interaction with maternal age [*F*_(1, 182)_ = 0.33, *ns*.]. There are no univariate effects of measurement point on the three well-being scales, nor are there any significant interactions of measurement points with SES-clusters, or measurement points with maternal age on the three scales. This means psychological well-being does apparently not change in neither one of the three SES clusters. There is, however, a marginally significant interaction between measurement points and SES clusters in the Satisfaction with Life Scale [*F*_(2, 182)_ = 2.97, *p* = 0.054, η_*p*_2 = 0.032]. The regression coefficients reveal significant differences in SWLS between the *more advantaged* cluster 3 and the *low education* cluster 2 [ß = −3.64, *t* = −2.75, *p* = 0.007], as well as between cluster 3 and the *lowest income* cluster 1 [ß = −4.851, *t* = −4.06, *p* < 0.001] at the first assessment. At the second assessment, only the difference between cluster 3 and cluster 1 remains significant [ß = −2.89, *t* = −2.47, *p* = 0.015]. This is due to apparent increases in SWLS in clusters 1 and 2, as opposed to an apparent decrease in SWLS in cluster 3 within 1 year. *Post-hoc* t-tests show that neither the decrease for cluster 3, nor the increases in clusters 1 and 2 are significant. Consequently, the overall pattern reflects stability in the three psychological well-being scales over a year in all three SES clusters (controlled for maternal age), and a stable influence of maternal age on psychological well-being.

## Discussion

The aim of this study was to examine the relation between SES and psychological well-being in a sample of Turkish immigrant mothers in Germany. We calculated the mothers' SES on the basis of mean net household income and maternal education. In comparison to the German majority population, this sample was poorer and less educated, and thus, overall of a lower SES. In order to uncover SES variance within this predominantly low SES sample, we decided to conduct a cluster analysis on family income and maternal education. The advantages of a cluster analysis is that it identifies subgroups in a particular sample providing information on their quality (i.e., fit indices). In contrast to, for example, a median split on the two variables after z-tranformation (to find two subgroups with lower and higher SES), a cluster analysis does not assume a linear relation of the two predictors but puts different sample-specific weights on the predictor variables. For our sample of Turkish immigrant mothers, the cluster analysis revealed a *lowest income* cluster (cluster 1), a *low-education* cluster (cluster 2), and a *more advantaged* cluster (cluster 3). Interestingly, these SES clusters differ in more relevant sociodemographic aspects than income and maternal education (see Table 4). Specifically, mothers in the *more advantaged* cluster as well as their partners were more likely to be gainfully employed and less likely to rely on social welfare benefits when compared to the other two clusters On the one hand, this confirms the relevance of income and education to calculate SES. On the other hand, this also reflects that income and education provide information on the social aspects of SES only indirectly. Although SES is generally assessed by means of financial and educational aspects, the social position of an individual or the participation in the workforce should also be considered.

In the second step of our analyses, we explored whether there are differences in psychological well-being between the three SES clusters. The pattern generally confirms the association between low SES and lower psychological well-being found in other studies for our low-SES sample of Turkish immigrant mothers. In a larger study, this is a study not focusing on Turkish immigrant mothers alone, the mothers reported on here would very possibly be grouped into one low-SES group. Our closer look at this low SES sample shows that there is well-being variance within this group. Although it has to be kept in mind that the three clusters we found are sample-specific and are thus not representative, it is interesting that the three clusters show significant differences in the scales we used to depict psychological well-being (CES-D-10, Satisfaction with Life Scale, and Daily Hassles). Here, mothers in the *more advantaged* cluster 3 had less depressive symptoms, a higher life satisfaction, and fewer daily hassles than mothers in clusters 1 and 2. Nonetheless, more than a quarter (28.1%) of the mothers in cluster 3 were identified as depressed when applying the respective criterion of the CES-D-10. This is less than in cluster 1 (39.4%) and cluster 2 (43.3%), but still almost twice as high as the depression prevalence rate for women in Germany, which is about 15% (Wittchen et al., 2010; Hapke, 2015). Considering that in the *low education cluster* 2, women are three times as likely to be depressed when compared to women of the Germany majority population, and that the *lowest income cluster* 1 yields a very similar depression rate, our data suggest that both low income and low education are risk factors for Turkish immigrant mothers in Germany. It has to be noted that the effects sizes in the MANOVAs are low. This is partially due to the sample size. A higher sample size would possibly have led to stronger effects. However, the low effect sizes may also be due to the specific sample itself and the many similarities the mothers have. We found SES variance in the sample but still the mothers do all have a low SES and it is thus not surprising that the three clusters do not differ as strongly on the three well-being scales as a medium-SES and a low-SES sample possibly would.

Our results further identify low SES as a risk factor for more daily hassles and for a reduced life satisfaction. Our cross-sectional data suggests that these effects are independent of the subjects' age. In the last step of our analyses, we inspected long-term effects of SES on psychological well-being with 1-year follow-up data. For the reduced sample of mothers who participated in the second assessment, we found a multivariate effect of maternal age. Regression coefficients revealed significant effects of mothers' age only for daily hassles at both measurement points. Across all clusters, older mothers were likely to report fewer daily hassles than younger mothers. The age of the children who participated in the main study varied between 5 and 15 years at the first assessment. Maternal age is significantly positively correlated with the age of the child tested [*r*_(*remaining*)_ = 0.282, *p* < 0.001]. We may thus interpret that the increased age-related independence of their children reduces the mothers' daily hassles, especially when taking into account that the mothers who participated in the longitudinal assessment had fewer children than mothers who dropped out for the second assessment.

Our results revealed a stable influence of SES on psychological well-being over a 1 year period. Although education and income had not been assessed a second time, we may assume there were hardly any changes regarding SES across the 12 months. We also found a marginally significant interaction between the SES clusters and the Satisfaction With Life Scale. At the first assessment, almost equally to the scores displayed in Figure 1, SWLS is highest in the *more advantaged cluster* 3 (*mean* = 26.85, *SD* = 6.42), and significantly lower in the *low education cluster* 2 (*mean* = 23.25, *SD* = 7.85) and *lowest income cluster* 1 (*mean* = 22.08, *SD* = 7.17). This pattern does not change at the second assessment (*mean* = 25.60, *SD* = 7.03 for cluster 3, *mean* = 24.23, *SD* = 7.20 for cluster 2, and *mean* = 22.79, *SD* = 6.49 for cluster 1). However, the insignificant decrease in SWLS means from the first to the second assessment in cluster 3, and the insignificant increases of SWLS means in cluster 1 and cluster 2 lead to the marginal significant interaction effect. The marginal increases in SWLS in clusters 1 and 2 might be an artifact of selective dropout. The most impaired mothers, i.e. those women who possibly considered a second study participation as too stressful, were most likely to not be reassessed. Mothers who dropped out after the first assessment had less education and more children, and they immigrated to Germany closer to adulthood. This increases the likelihood that they have had to face more challenges with language and the acculturation task, and thus more hassles in their daily lives. Therefore, it does not seem surprising that life satisfaction tendentially increased for clusters 1 and 2 within a year. Hence, it remains unclear whether the stable influence of low SES on poor psychological well-being is representative for the initial sample. Nonetheless, with more than 180 mothers, our follow-up sample still has a good sample size. Ideally, future research should be based on a larger initial sample size. In addition, a study on the association between SES and well-being among immigrant mothers would benefit from more information on the mother's cultural adaptation, such as language skills, perceived prejudices, or acculturation stress. One critical point of our data is that the entirety was based on mothers' self-reports. Ideally, other sources could have been added to validate our findings, although this would have complicated subject recruiting, and likely sample size, considerably.

It is also unclear to what extend the associations between SES and psychological well-being we found were influenced by factors we did not assess. Barger et al. (2009), who assessed SES based on education, income, employment status and wealth, found that social support, not SES, had the strongest influence on life satisfaction. We suppose that there would be similar effects, especially in Turkish (immigrant) families, as social support and family cohesion are of crucial importance for well-being in Turkish individuals (Kagitcibasi, 2007). Future studies should therefore also assess the participants' social and family support to uncover the relation of SES and psychological well-being. For repeated assessments, it would also be useful to reassess SES as this allows to inspect the relation of SES and well-being cross-sectionally for each assessment and the development of both SES and well-being across time.

Mothers' psychological well-being is important for their children's positive development, both for immigrant and non-immigrant children alike (Jaekel et al., 2015). Given that the women tested have a least one child, the high CES-D-10-scores we found, as well as the high daily hassles, and the relatively low life-satisfaction are alarming. Any improvement of the mothers' situation is likely to lead to better perspectives for their children as well (Goodman and Garber, 2017). We found SES, when based on income and education, to play an important role in maternal psychological well-being. This raises the question, to what extent mothers' well-being can be improved by a higher family income, and more investment into their education. As the possibilities here are limited and effortful (i.e., education needs time and dedication), social and psychological support respecting cultural and religious aspects could be helpful for Turkish immigrant mothers. Further studies should therefore focus on factors that support Turkish immigrants or other immigrant groups. Here, it has to be taken into account that first generation immigrants often have left family members behind in the country of origin, that they do not speak Germany very well, that they are likely to be poorly integrated into the host society, and that they quite possibly suffer acculturation stress (Spiegler et al., 2015). Further studies should therefore focus on factors that support immigrant parents' participation in the society by facilitating their access to education, language competencies, and consequently into the labor market as well.

For a sample of Turkish immigrant mothers in Germany, we were able to confirm the relation of SES and psychological well-being. Our initial sample size of 327 mothers allowed us to uncover SES variance in a generally low-SES sample. We were able to show that mothers with a slightly better education and a little more income were in a better situation regarding their psychological well-being, and that SES is a long-term influential factor on psychological well-being. However, psychological well-being was generally low in our sample, and in all likelihood this will impact the participants' children. Our results can thus also be understood as a claim to improve the situation for (Turkish) immigrant women and families in Germany and to facilitate their participation in society.

## Ethics statement

This study was carried out in accordance with the recommendations of the German Psychological Society (DGPs). The data collection procedure was approved by the Ethik-Kommission of the DGPs. All subjects gave written informed consent in accordance with the Declaration of Helsinki.

## Author contributions

IF pursued the data analyses and the manuscript writing. BL held the respective grant. She was involved in all stages the writing process of this paper.

### Conflict of interest statement

The authors declare that the research was conducted in the absence of any commercial or financial relationships that could be construed as a potential conflict of interest.

## Footnotes

^1^After Benjamini-Hochberg-correction for multiple comparison the p-values remain significant (*p* = 0.03 for SWLS, *p* = 0.025 for CES-D-10 and *p* = 0.025 for DH).

^2^The t-test reported here are all two-tailed.
